# Patterns of posterior ocular complications in myopic eyes of Indian population

**DOI:** 10.1038/s41598-018-29536-x

**Published:** 2018-09-12

**Authors:** Rohit Dhakal, Abhilash Goud, Raja Narayanan, Pavan K. Verkicharla

**Affiliations:** 1 Myopia Research Lab, Prof. Brien Holden Eye Research Centre, Kallam Anji Reddy Campus, L V Prasad Eye Institute, Hyderabad, 500034 Telangana India; 2 Brien Holden Institute of Optometry and Vision Sciences, Kallam Anji Reddy Campus, L V Prasad Eye Institute, Hyderabad, 500034 Telangana India; 3 Cinical Research, Prof. Brien Holden Eye Research Centre, Kallam Anji Reddy Campus, L V Prasad Eye Institute, Hyderabad, 500034 Telangana India; 4 Smt. Kanuri Santhamma Centre for Vitreo Retinal Diseases, Kallam Anji Reddy Campus, L V Prasad Eye Institute, Hyderabad, 500034 Telangana India

## Abstract

This is a retrospective study aimed to investigate the patterns of myopic fundus complications in Indian children and young adults. Electronic medical records of 29,592 patients, aged 10–40 years, who visited L V Prasad Eye Institute between 1^st^ January to 31^st^ December 2016 were analysed in the study. Data such as age, gender, refractive error and various pathologic lesions of posterior globe were considered for analysis. Among all the patients with different types of refractive errors, myopia was found in 47.4%, high myopia in 6.8% and pathologic myopia in 2.2%. There was no trend of the increased prevalence of pathologic myopia with increasing age, except for a significant difference between the children aged 10–15 years (2.7%) and those aged more than 15 years (>4%). . Although, the overall pattern of pathologic lesions was similar across different grades of myopia (2.5% in low myopes vs. 2.2% in severe myopes), lesions like staphyloma and retinal detachment increased with increasing degree of myopia. The proportion of pathologic lesions across different grades of myopia suggests the necessity for careful peripheral fundus examinations irrespective of the degree of myopia for better management and prognostic purposes.

## Introduction

Myopia has become an epidemic worldwide with steep increase in incidence of myopia in last few decades^1–4^. A meta-analysis published in 2017 shows the estimated pool prevalence of myopia to be 24.2% (95% CI, 3.3% to 44.8%) in children younger than 20 years and about 30% in adults older than 40 years in Asian population^1^. Globally, the prevalence of myopia has increased from 22.9% (uncertainty interval, 15.2–31.5%) in 2000 to 28.3% in 2010 (20.6–36.9%) and it is estimated to reach 49.8% by 2050^5^.

Continuous axial elongation of the eye leads to the over stretching of outer coats causing various pathologic changes such as tessellation of fundus, posterior staphyloma, optic disc changes, chorioretinal atrophy, lacquer cracks and choroidal neovascularisation^6^. Prevalence of pathologic myopia varies from 0.9% to 3.1% in Asians^7–10^ and 1.2% in Caucasians^11^. Vision impairment due to pathologic myopia is mostly irreversible^12^. The pathologic changes progress as the age increases and the associated vision threatening complications possibly increase the economic burden to the individual, family and country^13,14^. Around 5.8–7.8% of Europeans^15–18^, 4.5% of Latinos^19^ and 12.2–32.7% of the Japanese and Chinese population^20–22^ have low vision or blindness due to pathologic myopia.

Recent studies have shown the differences in structural and optical properties of eyes among different ethnicities^23–25^. As complications of myopia occur due to ocular stretching, and with the evidence that the ocular shape can be different among different ethnic groups^23^, it is possible that myopia related pathologic lesions may vary with different ethnic groups. Most of the studies investigated pathologic myopia in older adults, but not in children and young adults who when identified at an early age, can provide some insights into management options. Prevalence of myopia in Indian children a decade ago based on two publications was 4.1% in a rural population^26^ and 7.4% in urban population^27^ which increased to 13.1% in year 2015^28^. The prevalence and patterns of pathologic myopia manifestation in children and young adults in Indian population remains unclear. The aim of this study was to investigate patterns of high and pathologic myopia (at a tertiary eye care centres) in Indian children and young adults.

## Results

**Figure 1:**
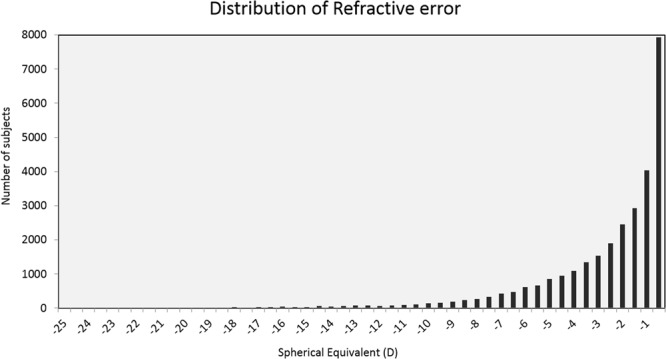
Distribution of myopes based on degree of refractive error (D-Dioptres).

The mean age of the individuals whose data was used in this study was 23.31 ± 7.21 years (range 10 to 40 years). Table 1 shows the distribution of individuals based on age, refractive error and gender. Majority of the individuals were in the age group of 21–30 years (47.85%) followed by 11–20 years (33.69%) and 31–40 years (18.46%). Majority of the eyes had mild myopia (65%) followed by moderate myopia (16.0%), high myopia (14.4%) and severe myopia (4.6%) as shown in Fig. 1. The number of males were 16,467 (55.64%).

**Table 1:** Distribution of myopes based on age, gender and refractive error.

	Number of subjects	Mild (−0.50 to −3.00 D)	Moderate (<−3.00 to −5.00 D)	High (<−5.00 to −10.00 D)	Severe (<−10.00 to −25.00 D)
		N (%)	N (%)	N (%)	N (%)
All	29,592	19,229 (65.0)	4,747 (16.0)	4,263 (14.4)	1,353 (4.6)
Age					
11–20	9,969	6,355 (63.7)	1,791 (18)	1,345 (13.5)	478 (4.8)
21–30	14,160	8,893 (62.8)	2,254 (15.9)	2,380 (16.8)	633 (4.5)
31–40	5,463	3,981 (72.9)	702 (12.9)	538 (9.8)	242 (4.4)
Gender					
Male	16,467	11,098 (67.4)	2,483 (15.1)	2,163 (13.1)	723 (4.4)
Female	13,125	8,131 (62.0)	2,264 (17.2)	2,100 (16.0)	630 (4.8)

Table 2 details the distribution of pathologic lesions on the posterior globe in different age groups and on gender basis. Among different pathologic lesions, lattice degeneration was the most common (2.65%, 95% CI- 2.5% to 2.8%) followed by tessellated fundus (2.07%, 95% CI-1.9% to 2.2%), WWOP (1.17%, 95% CI- 1% to 1.3%) and macular hole (1.16%, 95% CI- 1–1.3%). CNVM/CNV was least common and present only in 0.08% (95% CI- 0.1% to 0.1%) of total myopes. CRA, staphyloma and RD, which are among the leading sight threatening pathologic lesions, were found in 1.1% (95% CI- 1% to 1.2%), 0.15% (95% of CI- 0.1% to 0.2%) and 0.16% (95% CI- 0.1% to 0.2%) of total myopes, respectively. CRA, CNVM, posterior staphyloma and RD were more common in 36–40 years age group. There was no difference in pattern of pathologic lesions between males and females (P = 0.51, independent t-test) as shown in the Table 2. Among all myopes, 4.3% (95% CI- 4.1% to 4.5%) of them had pathologic myopia. Myopes in the age group of 21–25 years had the highest proportion of pathologic myopia lesions (4.9%, 95% CI- 4.4% to 5.4%) while the lowest proportion was seen in 11–15 years of age group (2.7%, 95% CI- 2.2% to 3.2%). WWP was present in less number of myopes (0.34%, 95% CI- 0.3% to 0.4%) compared to WWOP (1.17%, 95% CI- 1% to 1.3%).

**Table 2:** Patterns of various myopic fundus lesions among myopes according to age distribution and gender.

Age group (years)	Median (IQR)	Tessellation% (95% CI)	White without pressure	White with pressure	Lattice degeneration	Atrophy	Hole	CNVM/CNV	Staphyloma	RD	PM
Total	29,592	2.07% (1.9, 2.2)	1.17% (1, 1.3)	0.34% (0.3, 0.4)	2.65% (2.5, 2.8)	1.1% (1, 1.2)	1.16% (1, 1.3)	0.08% (0.1, 0.1)	0.15% (0.1, 0.2)	0.16% (0.1, 0.2)	4.3% (4.1, 4.5)
11–15 (N = 4338)	−2 (−1, −0.75)	3.3% (2.8, 3.9)	0.9% (0.6, 1.2)	0.2% (0.1, 0.3)	1.4% (1.1, 1.8)	1.1% (0.8, 1.4)	0.6% (0.4, 0.9)	0% (0, 0.1)	0.1% (0, 0.2)	0.1% (0, 0.2)	2.7% (2.2, 3.2)
16–20 (N = 5631)	−2 (−1, −4)	1.8% (1.5, 2.2)	1.6% (1.3, 1.9)	0.6% (0.4, 0.8)	2.5% (2.1, 2.9)	1.3% (1, 1.7)	1% (0.8, 1.3)	0.1% (0, 0.1)	0.2% (0.1, 0.3)	0.2% (0.1, 0.3)	4.2% (3.7, 4.8)
21–25 (N = 7898)	−2.25 (−1, −4.75)	1.9% (1.6, 2.2)	1.5% (1.3, 1.8)	0.5% (0.3, 0.6)	3.6% (3.1, 4)	0.8% (0.6, 1)	1.6% (1.3, 1.8)	0% (0, 0.1)	0.1% (0, 0.1)	0.2% (0.1, 0.3)	4.9% (4.4, 5.4)
26–30 (N = 6262)	−1.62 (−0.75, −0.75)	1.4% (1.1, 1.7)	1% (0.8, 1.3)	0.2% (0.1, 0.3)	3% (2.6, 3.4)	1% (0.8, 1.3)	1.3% (1, 1.5)	0.1% (0, 0.2)	0.1% (0, 0.2)	0.1% (0, 0.1)	4.6% (4.1, 5.2)
31–35 (N = 3156)	−1.5 (−0.75, −3.25)	2% (1.5, 2.4)	0.6% (0.3, 0.8)	0.2% (0, 0.3)	2.2% (1.7, 2.7)	1.4% (1, 1.8)	1% (0.7, 1.4)	0.1% (0, 0.2)	0.2% (0, 0.3)	0.2% (0, 0.3)	4.2% (3.5, 4.9)
36–40 (N = 2307)	−1.25 (−0.75, −2.75)	2.6% (1.9, 3.2)	0.5% (0.2, 0.8)	0.1% (0, 0.2)	2% (1.4, 2.5)	1.5% (1, 2)	1% (0.6, 1.5)	0.3% (0.1, 0.5)	0.4% (0.1, 0.6)	0.3% (0.1, 0.5)	4.6% (3.8, 5.5)
Gender											
Male (N = 16467)	2% (1.8, 2.3)	2.1% (1.9, 2.3)	1.2% (1, 1.4)	0.4% (0.3, 0.5)	2.6% (2.4, 2.9)	1.2% (1, 1.4)	1.2% (1, 1.4)	0.1% (0, 0.1)	0.2% (0.1, 0.2)	0.2% (0.1, 0.3)	4.4% (4.1, 4.8)
Female (N = 13125)	2% (1.8, 2.2)	2% (1.8, 2.2)	1.1% (0.9, 1.3)	0.3% (0.2, 0.4)	2.7% (2.4, 3)	1% (0.8, 1.1)	1.1% (0.9, 1.3)	0.1% (0, 0.1)	0.1% (0.1, 0.2)	0.1% (0.1, 0.2)	4.1% (3.8, 4.5)

In table, CNVM/CNV, RD and PM represent choroidal neovascular membrane/choroidal neovascularization, retinal detachment and pathological myopia, respectively.

Single eye analysis (only right eye analysed of the two eyes) showed 2.5% of myopes to have pathologic myopia. Whilst tessellation of fundus and lattice were observed in majority of patients, the occurrence of CNVM was much lower, a trend similar to what observed when both eyes were considered for analysis. Retinal holes, CRA, staphyloma and RD were present even in mild and moderate myopia groups as shown in Table 3. However, proportion of myopes having staphyloma and RD increased with the degree of myopia. Staphyloma was four times more common in severe myopes (0.4%) than in mild myopes (0.1%).

**Table 3:** Prevalence pattern of different pathologic myopia lesions among myopes in right eye.

Grades of Myopia	N	Tessellation % (95%CI)	White without pressure	White with pressure	Lattice degeneration	Atrophy	Hole	CNVM/CNV	Staphyloma	RD	PM
Total	29,529	2% (1.8, 2.2)	0.9% (0.8, 1)	0.3% (0.2, 0.3)	1.8% (1.7, 2)	0.3% (0.3, 0.4)	0.7% (0.6, 0.8)	0% (0, 0.1)	0.1% (0.1, 0.1)	0.1% (0.1, 0.1)	2.5% (2.3, 2.7)
<−0.50 to −3.00 D	20,053	2% (1.8, 2.2)	1% (0.9, 1.2)	0.3% (0.3, 0.4)	1.9% (1.7, 2.1)	0.3% (0.2, 0.4)	0.7% (0.6, 0.9)	0% (0, 0.1)	0.1% (0, 0.1)	0.1% (0.1, 0.1)	2.5% (2.3, 2.8)
<−3.00 to −5.00 D	4,567	1.5% (1.1, 1.8)	0.8% (0.6, 1.1)	0.2% (0.1, 0.3)	1.9% (1.5, 2.3)	0.4% (0.2, 0.5)	0.7% (0.5, 0.9)	0% (0, 0)	0.1% (0, 0.2)	0.1% (0, 0.1)	2.6% (2.1, 3.1)
<−5.00 to −10.00 D	3,860	2.2% (1.7, 2.6)	0.5% (0.3, 0.7)	0.1% (0, 0.2)	1.5% (1.1, 1.9)	0.4% (0.2, 0.6)	0.5% (0.3, 0.7)	0.1% (0, 0.2)	0.2% (0.1, 0.4)	0.2% (0.1, 0.4)	2.3% (1.9, 2.8)
<−10.00 to −25.00 D	1,112	2.4% (1.5, 3.3)	0.2% (−0.1, 0.4)	0.1% (−0.1, 0.3)	1.3% (0.6, 1.9)	0.3% (0, 0.6)	0.4% (0.1, 0.8)	0% (0, 0)	0.4% (0, 0.7)	0.2% (−0.1,0.4)	2.2% (1.3, 3)

In table, CNVM/CNV, RD and PM represent Choroidal Neovascular Membrane, Retinal Detachment and Pathological Myopia respectively.

## Discussion

Among different types of refractive errors, we found myopia in 47.4% of patients, high myopia in 6.8% and pathologic myopia in 2.2%. However, among only myopes, high myopia was found in 14.4% and pathologic myopia in 4.3%. Children between 10–15 years of age group had lowest proportion (2.7%) while adults between age group 25–30 years had highest proportion (4.9%) of pathologic myopia. Various pathologic lesions associated with myopia were found across all grades of myopia indicating that pathologic myopia lesions also exist in low grades of myopia (2.5% in low myopes vs. 2.2% in severe myopes). Lattice degeneration was the most frequent (2.7%) and CNVM/CNV was the least frequent lesion (0.08%) found in myopes.

Our result of 2.2% of pathologic myopia among patients with different types of refractive error lies within the prevalence reported in East Asian (Beijing Eye Study (3.1%)^9^, Shihpai Eye Study (3.0%)^7^ and Hisayama study (1.7%)^10^) and Caucasian populations (Blue Mountain Eye Study (1.2%)^12^). The Shihpai Eye Study conducted in Taiwan has defined the pathologic myopia taking into account both refractive error and axial length whereas our study has defined pathologic myopia based on fundus findings only. Direct comparison of our study results with Beijing, Shihpai, Hisayama and Blue Mountain studies may not be appropriate because of the following two reasons: Our study is a hospital based study while the other studies were population based study. Secondly, population included in these studies were elderly (≥49 years in Blue Mountain Eye Study, ≥65 years in Shihpai Eye Study, >40 years in Hisayama study and >40 years in Beijing Eye Study) while the current study included individuals within the age group of 10–40 years. In our study, the overall number of individuals with high myopia (<−5.0 D to −10.0 D) were found to be 6.8% which is higher than prevalence reported in Beijing Eye Study (3.3%), Hisayama study (5.7%) and Blue Mountain Eye Study (2.2%). In patients between 11–20 years of age, the prevalence of high myopia was found to be 2.15% which is significantly lower than the results reported by the study done in Singapore (7.3%)^29^ in adolescent children of age 14 years.

Beijing Eye Study showed the prevalence of staphyloma to be 1.6% and chorio-retinal atrophy to be 3.1%. In 2008, Lai *et al*.^30^ reported the prevalence of staphyloma to be 7.7%, chorio-retinal atrophy to be 2.7%, Lacquer crack to be 1.8% and Fuch’s spot to be 0.3% in the posterior pole. In the similar age group among myopic patients, the pattern of these pathologic lesion in our study is quite low, 0.15% of staphyloma and 1.1% of chorio-retinal atrophy. Proportionately, lattice degeneration stands highest (2.65%) among all types of pathologic lesions in Indian population.

Unlike other studies (Beijing, Shihpai and Blue Mountain Eye Study), the current study does not show a trend of increased prevalence of pathologic myopia with age, except for a significant difference between the ones aged 11–15 years and the remaining (2.7% in younger children vs. >4% in those aged >15 years). With the increasing age, several structural and functional changes occur in the posterior part of the eye^31,32^. A study conducted with OCT has shown thinning of retina with ageing^33^. As the age advances, lacquer cracks extend, increasing the area of atrophy in the macular area^34^. This could be the reason for increased prevalence of myopic maculopathy with age. In our study, the upper age limit is 40 years and this might have delivered lesser difference in proportion of patterns of various myopia complications between younger and older age group within 10–40 years. The results of current study also show no difference in the pattern of pathologic lesions with increase in grades of myopic refractive error. Beijing Eye Study had found prevalence of myopic retinopathy significantly increased with increasing refractive error, from 3.8% in eyes with <−4.0 D to 89.6% in eyes with at least −10.0 D. The Hisayama study also reported an increase in prevelence of pathologic lesions with increase in degree of myopia (myopic retinopathy increased from 0.3% in eyes with refractive error <−6.0 D to 36.8% in eyes with refractive error of at least −10.0 D). However, on analysing each pathologic lesion independently, we found that the proportion of staphyloma and RD increased with grades of myopia.

There was no difference in the pattern of pathologic myopia between male and female in our study, similar to the results from Blue Mountains and Beijing Eye Study. However, Hisayama study in Japanese elderly population reported female to have higher prevalence than males (2.2% vs. 1.2%). Similar outcomes were reported by Ito-Ohara *et al*. and Gozum *et al*. from Japan and Turkey respectively^35,36^.

The current study included 29,592 myopes for the analysis, which is so far, the highest number of patients enrolled for a study on patterns of pathologic myopia. This is one of the biggest strength of this study. In most of the East Asian and Caucasian studies, prevalence of pathologic myopia was seen in elderly population but we reported its prevalence in children and young adults. This study has some limitations. Firstly, our study is hospital based study where most of the patients’ visit only if they have some vision related problem. Secondly, the pattern of pathologic lesions were reported only among myopes. Thus, the results obtained in this specific age group may be an overestimated value i.e. proportion of people with myopia lesions. Considering that the few changes associated with pathologic myopia such as chorio-retinal atrophy and myopic CNV usually develop later in life, the use of data from 10 to 40 years age in this study does not give insights on overall proportion of myopia lesions in elderly population. Due to retrospective nature of the study, our study failed to analyse the axial length measurements and retinal Optical Coherence Tomography findings and classify the severity of the fundus changes based on the META-PM (META-analysis for Pathologic Myopia) study. Hence comparison of the results from this study with other studies and the generalisability of the outcomes of this study should be made with caution. Further population based studies are needed to understand the actual prevalence of pathologic myopia and its association with axial length. The relationship between various lesions of posterior globe with visual acuity and describing lesions based on its location were beyond the scope of this paper.

In conclusion, various pathologic lesions of myopia were found in 4.3% of myopes in our database. Although, the overall proportion of pathologic myopia was similar among all degrees of myopia, lesions like staphyloma and retinal detachment increased with increasing degree of myopia. The results from this study indicates the necessity for careful peripheral fundus examinations irrespective of degree of myopia for better management and prognostic purposes. Future studies are needed to understand the age appropriate strategies for management of pathologic myopia so that visually debilitating lesions like retinal detachment and CNVM/CNV can be prevented or strategically managed.

### Methodology

A retrospective analysis of patient’s electronic medical record (EMR), who visited any of the four tertiary centres of the L V Prasad Eye Institute (LVPEI), between 1^st^ January 2016 to 31^st^ December 2016, was conducted. Although, the four tertiary centres are located in four different geographical location of India, viz Bhubaneswar, Orissa; Visakhapatnam, Andhra Pradesh; Vijayawada, Andhra Pradesh; and Hyderabad, Telangana, the involved 29,592 patients came from different states panning from east to west and north to south India. Every single patient had signed informed general consent form prior to routine clinical examinations approving the use of their data for research purposes. The study was approved by institutional ethics committee of LVPEI, Hyderabad and procedures were in accordance to the tenets of the Declaration of Helsinki.

**Figure 2:**
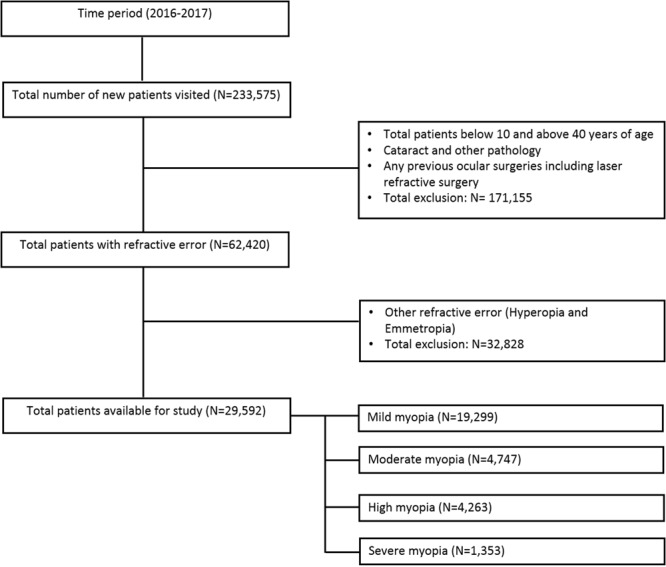
Flowchart of patient data pool at various levels of inclusion and exclusion stages.

A total of 233,575 patients visited for the first time during the chosen one-year study time period. To avoid the effect of any age-related changes on the refractive error like emmetropization or cataract, only patients above 10 years and below 40 years of age were included in the study. Any patients who either had media opacity or underwent any form of ocular surgery that could influence the refractive error were excluded from the study (N = 171,155) leaving behind 62,420 patients of which 29,592 were myopes. Figure 2 represents the flowchart of patient’s data pool at different level of inclusion and exclusion stages.

Demographic data such as age and gender, and clinical data such as refractive error and retinal signs as documented in EMR by treating ophthalmologist using indirect ophthalmoscope and slit lamp biomicroscope were collected. Medical records were reviewed to identify various pathologic myopia lesions, namely tessellation of fundus, white without pressure (WWOP), white with pressure (WWP), lattice degeneration, chorio-retinal atrophy (CRA), retinal holes, choroidal neovascularisation (CNVM/CNV), posterior staphyloma and retinal detachment (RD). Considering that the CNVM/CNV can also occur due to causes other than myopia, viz. idiopathic CNVM and punctate inner choroidopathy, care was taken to exclude such findings. Pathologic myopia was defined as the presence of any one of the following retinal signs: lattice degeneration, CRA, retinal holes, CNVM/CNV, posterior staphyloma or RD^16^.

The prevalence of high myopia and pathologic myopia based on age, gender and grades of myopia was expressed in percentage (%). Data of both the eyes were collected and if above mentioned signs were present in at least one of the eye, then patient was labelled to have pathologic myopia lesion. To understand the patterns of pathologic lesions based on refractive error, data of only right eye were analysed. Myopia was divided into 4 categories i.e. mild, moderate, high and sever where mild myopia was defined as spherical equivalent (SE) of <−0.50 D to −3.00 D, moderate myopia as <−3.00 D to −5.00 D, high myopia as <−5.00 D to −10.00 D and severe myopia as <−10.00 D and above^5^.
